# Characterization of a Bacterium Isolated from Hydrolyzed Instant Sea Cucumber *Apostichopus japonicus* Using Whole-Genome Sequencing and Metabolomics

**DOI:** 10.3390/foods13223662

**Published:** 2024-11-17

**Authors:** Xin Luo, Zhixuan Zhang, Zhangyi Zheng, Wenwen Zhang, Tinghong Ming, Lefei Jiao, Xiurong Su, Jiajie Xu, Fei Kong

**Affiliations:** 1 School of Marine Science, Ningbo University, Ningbo 315211, China; sanjinjin1107@126.com (X.L.); zhixuan27@foxmail.com (Z.Z.); 2211130073@nbu.edu.cn (Z.Z.); 2211130071@nbu.edu.cn (W.Z.); mingtinghong@nbu.edu.cn (T.M.); jiaolefei@nbu.edu.cn (L.J.); suxiurong_public@163.com (X.S.); 2 Microbial Development and Metabolic Engineering Laboratory, Ningbo University, Ningbo 315211, China; 3 Collaborative Innovation Center for Zhejiang Marine High-Efficiency and Healthy Aquaculture, Ningbo 315211, China

**Keywords:** *Apostichopus japonicus*, autolysis, microorganism

## Abstract

Autolysis in the sea cucumber *Apostichopus japonicus* is typically triggered by degradation caused by microorganisms within their bodies. However, information on this topic remains limited. Recently, we isolated and purified a bacterial strain from hydrolyzed instant sea cucumber samples. To investigate its potential role in the autolysis process, this study employed whole-genome sequencing and metabolomics to explore its genetic and metabolic characteristics. The identified strain was classified as *Lysinibacillus xylanilyticus* and designated with the number XL-2024. Its genome size is 5,075,210 bp with a GC content of 37.33%, encoding 5275 genes. Functional database comparisons revealed that the protein-coding genes were distributed among glucose metabolism hydrolase, metal hydrolase, lysozyme, cell wall hydrolase, and CAZymes. Compared to 20 closely related strains, *L. xylanilyticus* XL-2024 shared 1502 core homologous genes and had 707 specific genes. These specific genes were mainly involved in the carbohydrate metabolism pathway and exhibited glycosyl bond hydrolase activity. Metabolomic analysis showed that *L. xlanilyticus* XL-2024 produced several metabolites related to polysaccharide degradation, including peptidase, glucanase, and pectinase. Additionally, the presence of antibacterial metabolites such as propionic acid and ginkgo acid among its metabolites may enhance the stability of the sea cucumber hydrolysate. In summary, *L. xylanilyticus* XL-2024 may play a pivotal role in the autolysis of *A. japonicus*. The results of this study provide a strong foundation for understanding how to prevent autolysis in *A. japonicus* and for better utilizing *L. xylanilyticus* XL-2024.

## 1. Introduction

*Apostichopus japonicus* is a species of echinoderm belonging to the family of sea cucumbers, known for its high nutritional and economic value [1]. However, this species is highly susceptible to autolysis, primarily due to the autolytic enzymes that are activated during processing and transportation [2]. To address this issue, various technologies have been developed to create instant sea cucumber products that effectively eliminate these autolytic enzymes. Nevertheless, instant sea cucumbers still experience instability issues, such as softening and stickiness, which lead to tissue degradation and damage when stored at room or low temperatures [3]. The key components in the body wall of instant sea cucumbers are collagen [4] and polysaccharides [5]. Therefore, it is believed that the instability of sea cucumbers may be linked to the degradation of these two substances [6].

More specifically, *A. japonicus* comprises approximately 40–65% protein and 10% anionic polysaccharides on a dry-weight basis, primarily including sulfated fucan (also known as fucoidan) and fucosylated chondroitin sulfate [7]. The monosaccharide composition of sea cucumber polysaccharides identified so far includes N-acetylgalactosamine, galactosamine, galactose, fucose, glucosamine, glucuronic acid, and mannose [8,9]. The glycosidic bonding is characterized by *α*-1,3, *α*-1,4 linkages [10,11]. Correspondingly, carbohydrate-active enzymes (CAZymes) play a crucial role in glycoside bond degradation, modification, and formation [12]. These enzymes have been classified into six families: Glycosyl Hydrolases (GHs), Glycosyl Transferases (GTs), Polysaccharide Lyases (PLs), Carbohydrate Esterases (CEs), Carbohydrate-Binding Modules (CBMs), and Auxiliary Activities (AAs) [13]. GHs catalyze the hydrolysis of the glycosidic linkage in polysaccharides (e.g., cellulose, chitin) and oligosaccharides (e.g., cellobiose, chitobiose) [14]. GTs are involved in the biosynthesis of glycosidic bonds from phospho-activated sugar donors [15]. PLs cleave the glycosidic bonds of uronic acid-containing polysaccharides via a *β*-elimination mechanism [16]. CEs are responsible for removing the ester-based modifications present in mono-, oligo-, and polysaccharides, which helps facilitate the action of GHs on complex polysaccharides [17]. CBMs lack intrinsic enzymatic activity; however, they support the action of these enzymes by enabling extended interactions with the substrate [18]. Notably, microbes can produce a diverse array of the CAZymes mentioned above, and the types and activities of these enzymes are closely related to the characteristics of CAZyme genes present in the bacterial genome [19]. These findings indicate that microorganisms may play a critical role in the autolysis of *A. japonicus*. However, such information on this topic is still scarce.

Recently, we discovered that instant *A. japonicus* undergoes hydrolysis after exposure to ^60^Co irradiation. By cultivating the hydrolysate, we successfully isolated and purified a specific bacterium. To investigate its potential role in the autolysis of *A. japonicus*, this study employed whole-genome sequencing and metabolomics to explore the genetic and metabolic characteristics of the bacterium. The results will provide a solid foundation for understanding how to prevent the autolysis in *A. japonicus* and offer insights into the potential utilization of this specific bacterium.

## 2. Materials and Methods

### 2.1. Isolation and Culture of the Bacterium

The vacuum-packaged, ready-to-eat *A. japonicus* samples from the East China Sea exhibited liquefaction after undergoing ^60^Co irradiation sterilization. Notably, these samples showed no signs of decay from 2007 to 2021. To investigate the liquefaction, a portion of the liquid from the affected samples was transferred to nutrient broth (NB) medium containing peptone (10.0 g/L), beef extract (3.0 g/L), and NaCl (5.0 g/L). The mixture was incubated overnight at 37 °C in a constant temperature shaker until turbidity was observed. Subsequently, the turbid broth was streaked onto nutrient agar plates to isolate and purify the colonies. This process yielded a pure culture strain, which was subjected to whole-genome sequencing.

### 2.2. Whole-Genome Sequencing and Assembly

Genomic DNA was extracted using the SDS method [20]. The extracted DNA was analyzed via agarose gel electrophoresis and quantified by using the Qubit^®^ 2.0 Fluorometer (Thermo Fisher Scientific, Waltham, MA, USA). The NEBNext^®^ Ultra™ DNA Library Prep Kit for Illumina (NEB, Ipswich, MA, USA) was utilized for sequencing library preparation, with index codes added to associate the sequences with each sample. The whole genome of this strain was sequenced using the Illumina NovaSeq PE150 platform at LC-Bio Technology Co., Ltd., in Hang Zhou, Zhejiang Province, China.

The raw sequencing data were initially filtered using readfq (version 10) to obtain clean data. Bacterial genome assembly standards generally include N50 and N90 lengths (indicating continuity), genome completeness, and low contamination rates to ensure high-quality results, providing a solid foundation for the further analysis. For the genome of *L. xylanilyticus* XL-2024, after preprocessing, clean data were assembled using various software tools, including SOAP de novo (version 2.04) [21,22], SPAdes (version 3.13.0) [23], and ABySS (version 2.2.3) [24]. Subsequently, CISA (version 1.3) [25] software was employed for integration. The preliminary assembly results were optimized, and the gaps were filled using gapclose (version 1.12) to obtain the final assembly. Fragments shorter than 500 bp were filtered out, and the contaminated samples were decontaminated as needed. The subsequent gene predictions were performed following assessment and statistical analysis.

### 2.3. Gene Function Analysis

The protein sequences of the predicted genes were compared against various functional databases using Diamond alignment, with an evaluation threshold of ≤1 × 10^−5^. For each sequence alignment, the highest scoring result (default identity ≥ 40%, coverage ≥ 40%) was selected for functional annotation. The databases used for annotation included the Non-Redundant Protein Database (NR), Cluster of Orthologous Groups (COG), Gene Ontology (GO), Kyoto Encyclopedia of Genes and Genomes (KEGG), CAZymes Database (CAZy), Transporter Classification Database (TCDB) [26], Pfam, and Swiss-Prots.

The secretory proteins were predicted via the SignalP (version 4.1), while the prediction of Type I-VII proteins secreted by the pathogenic bacteria was performed using EffectiveT3 software (Version 1.0.1) [27]. Additionally, we analyzed the secondary metabolism gene clusters using antiSMASH. The pathogenicity and drug resistance analyses were conducted for pathogenic bacteria. Virulence or pathogenicity analysis was carried out using the Pathogen Host Interactions Database (PHI) [28], Virulence Factors of Pathogenic Bacteria (VFDB) [29], Antibiotic Resistance Genes Database (ARDB), and Comprehensive Antibiotic Research Database (CARD).

### 2.4. Gene Family Analysis

First, the protein sequences of the reference genomes were downloaded and filtered based on their length, excluding sequences shorter than 50 amino acids. All the protein sequences intended for the analysis were merged into a single file, which served as the dataset for database construction. This database was then used as a query to perform an all-VS-all blast analysis, with the alignment threshold set to 1 × 10^−10^. The resulting sequence alignment was further processed using Orthotics software (version 2.0.8) [30]. The alignment length threshold was set to 70%, and gene families were clustered using MCL, with an inflation (I) parameter set to 1.5.

### 2.5. Extraction of Metabolites

The activated *L. xylanilyticus* XL-2024 bacteria were incubated in nutrient broth liquid medium for 12 h under aerobic conditions at 37 °C. Subsequently, the bacteria were spread on nutritional broth agar solid medium and incubated at 37 °C for 24 h under aerobic conditions. The bacteria were then collected and extracted using a 50% methanol buffer. For the extraction, 20 μL of the bacterial sample was mixed with 120 μL of precooled 50% methanol, vortexed for 1 min, and incubated at room temperature for 10 min. The extraction mixture was stored overnight at −20 °C. After centrifugation at 4000× *g* for 20 min, the supernatants were transferred into new 96-well plates and stored at −80 °C until the UHPLC-MS analysis.

### 2.6. UHPLC-MS Analysis

The UHPLC-MS analysis was performed using a UHPLC system (SCIEX, Framingham, MA, USA) coupled with a mass spectrometer (SCIEX, Framingham, MA, USA). An ACQUITY UPLC HSS T3 column (100 × 2.1 mm, 1.8 µm, Waters, Milford, MA, USA) was used for the reversed-phase separation. The column oven was maintained at 35 °C, and the flow rate was set to 0.4 mL/min. The mobile phase consisted of solvent A (0.1% formic acid in water) and solvent B (0.1% formic acid in acetonitrile). The solvent gradient was programmed as follows: 0–0.5 min, 5% B; 0.5–7 min, 5% to 100% B; 7–8 min, 100% B; 8–8.1 min, 100% to 5% B; 8.1–10 min, 5% B. The injection volume for each sample was 4 µL.

The raw data files generated by UHPLC-MS were processed using the XCMS, CAMERA, and metaX toolbox implemented in R software (Version 3.6.2). Each ion was identified by combining the retention time (RT) and *m*/*z* data. The metabolites were annotated using the KEGG and HMDB database, matching the exact molecular mass data (*m*/*z*) of the samples with those in the database. The peak intensity data were further preprocessed using the metaX tool. The features detected in fewer than 50% of the quality control (QC) samples or 80% of the biological samples were removed, and the missing values in the remaining peaks were imputed using the k-nearest neighbor algorithm to improve the data quality. Principal component analysis (PCA) was performed on the preprocessed dataset to detect outliers and evaluate batch effects.

## 3. Results

### 3.1. Genomic Characteristic of the Purified Bacterium

The strain was identified as *L. xylanilyticus* XL-2024 through the Non-Redundant Protein Database (NR) of whole-genome sequencing. Its genomic size was 1115 Mb of the raw data. After removing the adapters and low-quality reads, we obtained 1000 Mb of valid data, with Q20 and Q30 scores of 97.29% and 92.00%, respectively (Table 1). These results demonstrated that the data quality was sufficient for the subsequent analysis. Using K-mer statistics with a 15-mer length, a total of 265,881,182 k-mers were extracted from the reads (Table 2). Based on the valid data, genome assembly was performed with no gaps, resulting in a total of 47 scaffolds. The assembled genome had a total length of 5,075,210 bp, with N50 and N90 lengths of 217,930 bp and 78,763 bp, respectively (Table 3). Subsequently, the GeneMarkS program (version 4.17) predicted a GC content of 37.33%, with 5275 coding genes and an average gene length of 782 bp (Table 4).

In addition, we identified 567 tandem repeats (TRs) in the *L. xylanilyticus* XL-2024 genome, with the repeat sizes ranging from 3 to 374 bp. The total length of these TRs was 56,515 bp, accounting for 1.11% of the *L. xylanilyticus* XL-2024 whole genome sequence (Table 5). Furthermore, 253 non-coding RNAs (ncRNA) were identified in the genome, including 79 tRNAs, 14 rRNAs, and 160 sRNAs (Table 6). The genome also contained six genomic islands (GIs) with a total length of 70,783 bp and an average length of 11,797 bp. Additionally, three prophages were identified, with a total length of 76,663 bp and an average length of 25,554.3 bp. Moreover, 28 CRISPR elements were predicted, with a total length of 6278 bp and an average length of 224.214 bp. The assembly details and genome characteristics are summarized in Table 6.

### 3.2. Functional Annotation of the Purified Bacterium

**Figure 1:**
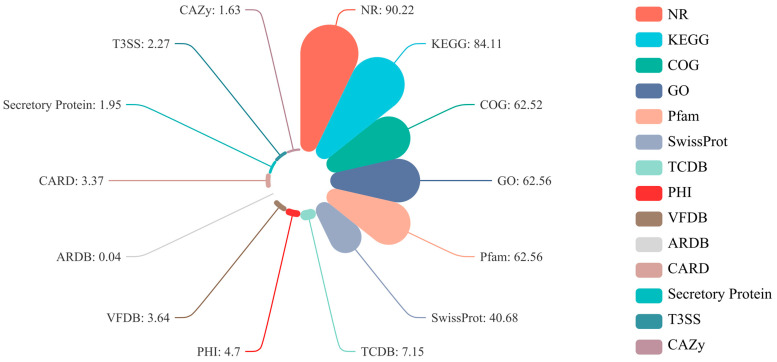
Percentage (%) of genes functionally annotated in various databases within the *L. xylanilyticus* XL-2024 genome.

The universal function database successfully labeled 5275 protein-coding genes in the genome of *L. xylanilyticus* XL-2024. As shown in Figure 1, the NR database had the highest number of functionally annotated genes, with 4759 genes (Table S1), accounting for 90.22% of the total number of genes. This was followed by the KEGG database, which annotated 4437 genes, accounting for 84.11% of the total number of genes. The GO and Pfam databases each annotated 3300 genes, representing 62.56% of the total number of genes. The COG database annotated 3298 genes, accounting for 62.52%. In the CAZy database, 86 genes were annotated, accounting for 1.63% of the total. Notably, the number of genes with an effective T3SS was 120, accounting for 2.27% of the total coding genes.

**Figure 2:**
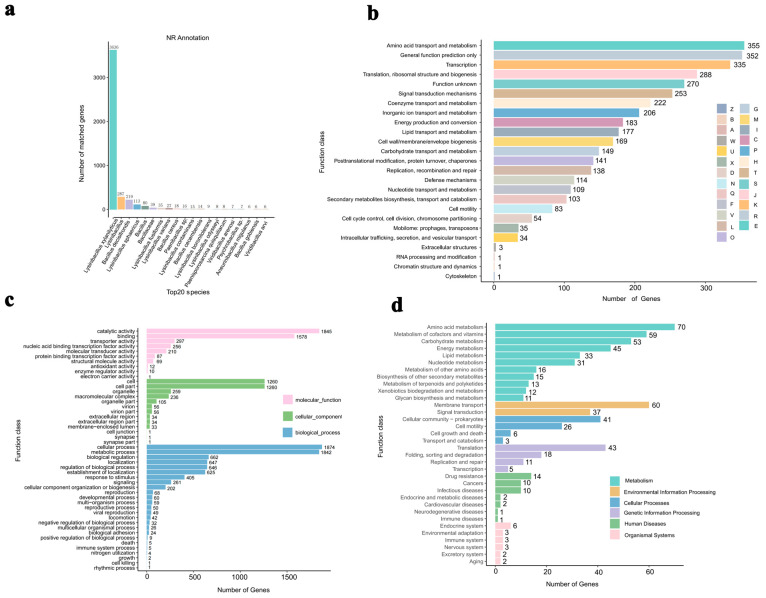
Annotation and functional classification of genes in NR (**a**), COG (**b**), GO (**c**), and KEGG (**d**) databases within the *L. xylanilyticus* XL-2024 genome.

As depicted in Figure 2a, *L. xylanilyticus* XL-2024 has the largest number of protein-coding genes annotated in the NR database, with 3636 genes. These genes were primarily distributed among glucose metabolism hydrolases, metal hydrolases, lysozyme, and cell wall hydrolases (Table S2). Specifically, the genes annotated as glucose metabolism hydrolases included GM001027, GM001892, GM000558, GM001264, GM004360, and GM003654, for a total of six genes. Metal hydrolases included GM000747, GM000976, GM001253, GM001255, GM000993, GM001010, GM001191, GM003620, GM005159, GM002286, GM000777, GM003563, GM004187, and GM003726. Lysozyme-related genes included GM003063, GM004272, GM004612, and GM004811. Cell wall hydrolases included GM004137, GM005221, GM003748, GM002302, and GM004327. Furthermore, the COG functional annotation of the *L. xylanilyticus* XL-2024 coding sequence identified 25 functional groups (Figure 2b). Notably, a significant proportion of the genes were associated with “amino acid transport and metabolism” (355 genes, 10.76%), “general function prediction only” (352 genes, 10.67%), and “transcription” (335 genes, 10.16%). A total of 288 genes were related to “translation, ribosomal structure, and biogenesis”. Additionally, 270 coding genes exhibited “unknown gene functions”, indicating that there are still many gene functions to be explored within this genome.

In addition, the protein sequences encoded by *L. xylanilyticus* XL-2024 have been annotated in the GO database (Figure 2c). The 3300 coding genes were primarily distributed among 48 functional categories, which were further divided into three main categories: “biological processes”, “cellular component”, and “molecular function”. Within the “biological process” category, 1874 and 1842 genes were annotated as “cellular process” and “metabolic process”, respectively. Regarding the “cellular component”, 1260 genes were annotated as “cell and cell parts”. In terms of “molecular function”, “catalytic activity” and “binding” were the most prevalent annotations (Figure 2c). As shown in Figure 2d, the KEGG annotation results indicated that most of the functional genes were enriched in four main categories: “metabolism”, “environmental information processing”, “genetic information processing”, and “cellular processes”, with 358, 97, 77, and 76 genes annotated in each category, respectively. Notably, 53 genes were annotated as “carbohydrate metabolic pathways”. Among these, eleven genes were implicated in “glycolysis”, two in “galactose metabolism”, and three in “fructose and mannose metabolism” (Figure 2d).

Notably, a total of 86 CAZy family genes were identified in the genome of *L. xylanilyticus* XL-2024 (Table 7). Among those, 57 genes were dedicated to polysaccharide degradation, comprising thirty CBMs, nineteen GHs, seven CEs, and one PL. A notable feature within the degradation-related genes is the presence of 26 CBM50 genes, which are linked with various enzymes from the GH families 18, 19, 23, 24, 28, and 73. These enzymes are primarily involved in the breakdown of chitin and peptidoglycan. Specifically, GH23 also exhibits lysozyme type G activity, indicating its role in bacterial cell wall degradation. The CBM50 module is particularly versatile in enzymes targeting peptidoglycans, including peptidases and amidases. Additionally, enzymes associated with the GH13 family (specifically GH13_3, GH13_11, and GH13) are identified as possessing pullulanase activity, which is important for the breakdown of certain polysaccharides. Other CBMs and GHs are involved in binding specific sugars; for instance, CBM48 is linked to glycogen binding, while CBM32 and GH1 are associated with the binding of galactose and lactose, respectively. The gene repertoire also includes five additional genes (CBM13, CBM5, CE4, CE9, and PL9_2) that contribute to cell wall degradation, targeting substances like xylanase, chitin, and pectate. Moreover, the genome contains 29 glycosyltransferases (GTs), which are primarily predicted to function as sucrose synthase, cellulose synthase, *β*-glucuronosyltransferase, and *β*-N-acetylmannuronosyl transferase. These GTs are likely involved in synthesizing nucleotide sugar precursors, such as UDP-rhamnose, UDP-glucose, and GDP-mannose. Overall, these findings highlight the rich enzymatic toolkit of *L. xylanilyticus* XL-2024, allowing it to effectively degrade various polysaccharides, chitin, and peptides, while also exhibiting lysozyme activities that facilitate the breakdown of bacterial cell walls.

In addition, the analysis of the *L. xylanilyticus* XL-2024 genome revealed a comprehensive array of transporters, as shown in Figure S1. A total of 377 transporters were identified and classified according to the TCDB. These transporters were classified according to the primary and secondary levels of the TCDB transfer system. In the primary level classification (Figure S1a), the largest group was “Primary Active Transporters”, comprising 205 transporters. The second-largest group was “Electrochemical Potential-driven Transporters”, with 122 transporters. Additionally, 23 transporters were annotated as “Channels/Pores”. In the secondary-level classification (Figure S1b), 186 transporters were annotated as “P-P-bond-hydrolysis-driven transporters”. The second-largest group was “Porters (uniporters, symporters, antiporters)”, which includes 122 genes. In contrast, the category with the fewest genes was “Beta-Barrel Porins”, with only one gene. Furthermore, the annotations from the Pfam database indicated that the “P-loop containing nucleoside triphosphate hydrolase superfamily” had the highest number of gene annotations, with 1664 genes belonging to this functional category (Figure S2). Following that, “FAD/NAD(P)-Binding Rossmann fold superfamily” and “Helix-turn-helix clan” have relatively more genes, which have 1034 and 865 genes, respectively.

### 3.3. Virulence Factor Analysis of the Purified Bacterium

The *L. xylanilyticus* XL-2024 genome was subjected to analysis using the Virulence Factors Database (VFDB), which resulted in the annotation of 192 virulence factor genes. With a screening condition of identity ≥ 70%, eight virulence genes were identified in the genome of *L. xylanilyticus* XL-2024 (Table S3). Among these, three genes were not annotated, two were related to bacterial adhesion, two were associated with stress survival, one was involved in immune modulation, and none were linked to toxin production.

### 3.4. Gene Family Analysis of the Purified Bacterium

**Figure 3:**
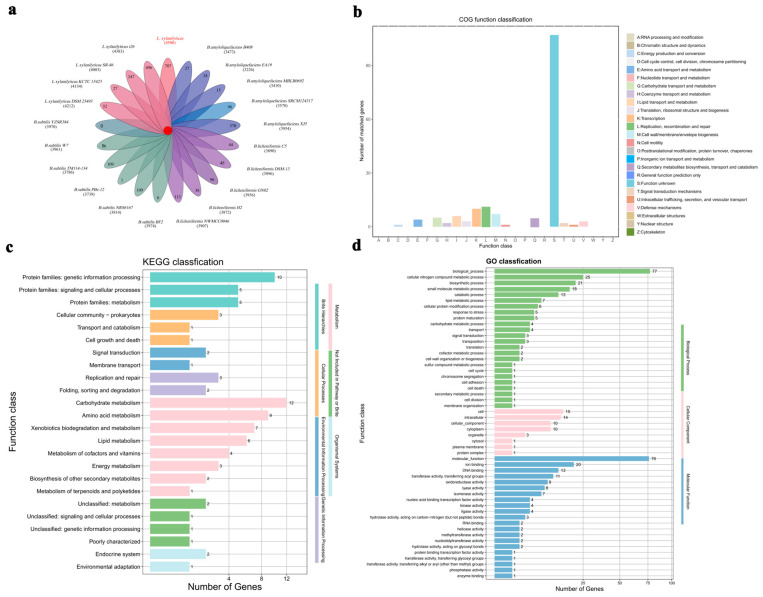
Pan analysis of the genome of *L. xylanilyticus* XL-2024 and 20 other similar-type strains. (**a**) Flower plot displaying the core and specific proteins in 21 strains. Each petal represents a strain, with the number of core proteins shown in the center. The non-overlapping sections indicate the number of strain-specific proteins, and the strain name is next to each petal. The *L. xylanilyticus* (XL-2024) is highlighted in red. (**b**) COG function classification of *L. xylanilyticus* XL-2024-specific genes. (**c**) KEGG pathway classification of *L. xylanilyticus* XL-2024-specific genes. (**d**) GO function classification of *L. xylanilyticus* XL-2024-specific genes.

A total of 4506 genes were annotated, including 1502 common genes and 707 specific genes (Figure 3a) when compared to those in other species within the *Lysinibacillus* genus. According to the COG database (Figure 3b), 13 genes were classified under “Replication, recombination, and repair (L)”, and 10 genes were annotated as “Transcription”. A substantial number of genes were designated as “function unknown (S)”. As shown in Figure 3c, the KEGG pathway annotation results showed that 12 genes were enriched in “Carbohydrate metabolism”, and 10 genes were enriched in “Protein families: genetic information processing”. The GO analysis results revealed that “biological processes” had the highest number of enriched genes, with 205 genes, followed by “molecular functions” with 174 genes, and “cellular components” with 55 genes (Figure 3d). Specifically, nine genes exhibited “oxidoreductase activity”, three genes had “hydrolase activity acting on carbon-nitrogen (but not peptide) bonds”, two genes had “helicase activity”, and two genes had “hydrolase activity acting on glycosyl bonds” (Figure 3d). The TCDB primary classification map indicated that three genes were enriched in “Electrochemical Potential-driven Transporters” (Figure S3a). In the PHI database, five genes were enriched in “reduced virulence”, and three genes were associated with “unaffected pathogenicity” (Figure S3b).

### 3.5. Metabolomic Analysis of the Purified Bacterium

As shown in Figure S4, the TIC graphs of the QC samples were highly overlapped in positive and negative ion modes, indicating the stable instrument performance and the reliability of the obtained data.

**Figure 4:**
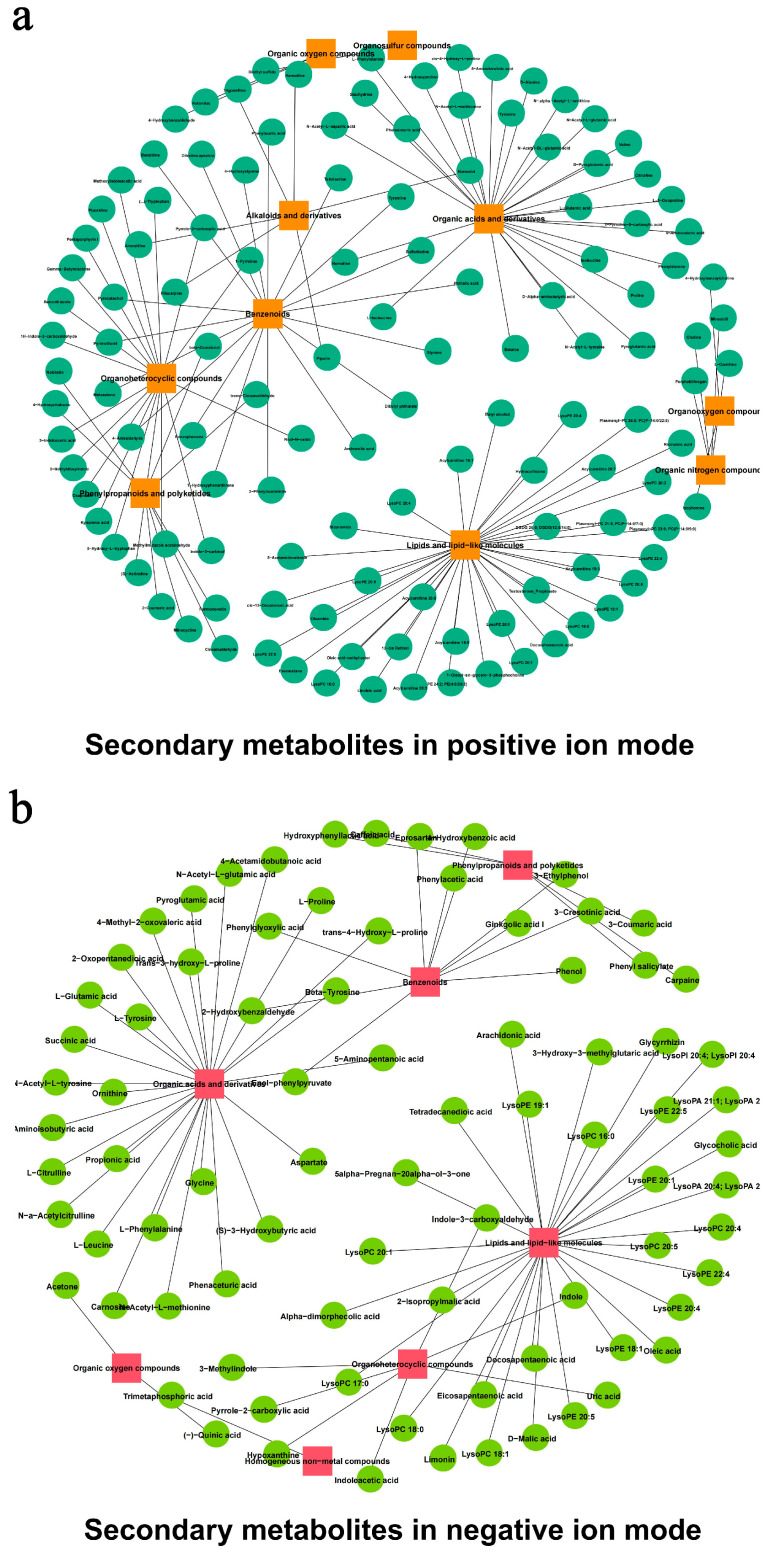
Metabolite analysis of *L. xylanilyticus* XL-2024 in the positive ion mode (**a**) and negative ion mode (**b**), respectively.

In the positive ion mode (Table S4), 336 metabolites were identified. Among them, the most numerous were “Organic oxygen compounds” (141), followed by “Organoheterocyclic compounds” (63), “Lipids and lipid-like molecules” (62), “Benzenoids” (46), “Phenylpropanoids and polyketides” (17), and “Alkaloids and derivatives” (7), respectively. In the negative ion mode (Table S4), 184 metabolites were identified, with “Organic acids and derivatives” being the most abundant (74 species), followed by “lipids and lipid-like molecules” (61 species). “Organoheterocyclic compounds” (twenty-one species), “Benzenoids” (seventeen species), “Phenylpropanoids and polyketides” (five species), “Organic oxygen compounds”, and “Homogeneous non-metal compounds” were the least abundant, with four, and two species, respectively. Some specific metabolites were detected in both modes, including “Phenyl salicylate”, “Caffeic acid”, and various “Organoheterocyclic compounds” such as Indoleacetic acid, Indole-3-carboxyaldehyde, and Hypoxanthine. Additionally, among the “Organic acids and derivatives”, Carnosine, Propionic acid, L-Tyrosine, Succinic acid, and Ornithine were identified. The “Lipids and lipid-like molecules” included Arachidonic acid, Oleic acid, and D-Malic acid, while the “Benzenoids” encompassed Ginkgolic acid I, Phenylacetic acid, and Phenol (Figure 4). Other metabolites such as glutamic acid, proline, fatty acids, polysaccharides, glycosides, and antibiotics were also identified.

### 3.6. Metabolic Pathway Analysis and Enrichment Analysis

**Figure 5:**
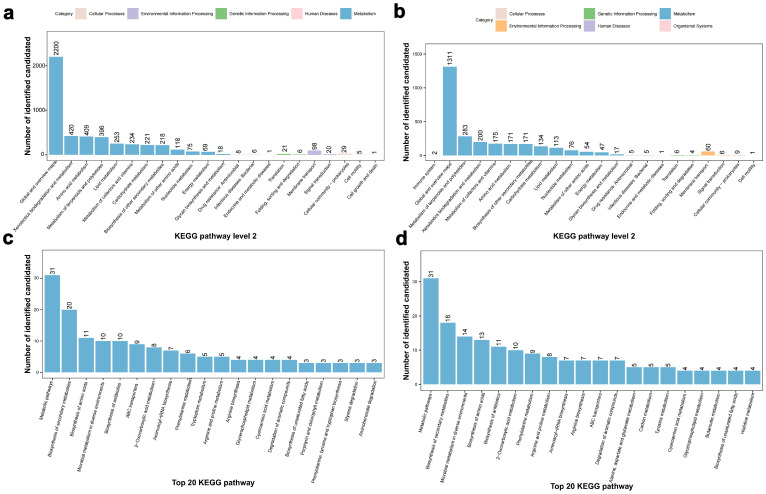
KEGG functional annotation analysis of secondary metabolites of *L. xylanilyticus* XL-2024. (**a**) KEGG pathway classification diagram in the positive ion mode. (**b**) KEGG pathway classification diagram in the negative ion mode. (**c**) Top 20 KEGG pathway classification diagram in the positive ion mode. (**d**) Top 20 KEGG pathway classification diagram in the negative ion mode.

Among the KEGG primary metabolic pathways, the “Global and overview maps” category had the largest number of enriched genes in both the positive and negative ion modes, with 2200 and 1311 genes, respectively (Figure 5). In the positive ion mode, the number of genes enriched in “Xenobiotics biodegradation and metabolism”, “Amino acid metabolism”, “Metabolism of terpenoids and polyketides”, “Lipid metabolism”, and “Metabolism of cofactors and vitamins” were 420, 409, 396, 253, and 234, respectively (Figure 5a). In the negative ion mode, the number of genes enriched in “Metabolism of terpenoids and polyketides”, “Xenobiotics biodegradation and metabolism”, “Metabolism of cofactors and vitamins”, “Amino acid metabolism”, and “Biosynthesis of other secondary metabolites” were 283, 200, 175, 171, and 171, respectively (Figure 5b). Among the top 20 secondary metabolic pathways of KEGG, in the positive ion mode, the number of enriched genes in the “Metabolic pathways”, “Biosynthesis of secondary metabolites”, “Biosynthesis of amino acids”, and “Microbial metabolism in diverse environments” were 31, 20, 11, and 10, respectively (Figure 5c). In the negative ion mode, the “Metabolic pathways”, “Biosynthesis of secondary metabolites”, “Microbial metabolism in diverse environments”, and “Biosynthesis of amino acids” were enriched with 31, 18, 14, and 13 genes, respectively (Figure 5d).

## 4. Discussion

*A. japonicus* is highly nutritious. However, due to autolysis, it typically appears in a ready-to-eat state when presented to the public [31]. In the present study, the dissolution of instant sea cucumber was observed after ^60^Co irradiation, and a bacterium was found in its hydrolysate. Whole-genome sequencing and the comparative analysis of gene families were performed to identify and analyze its genetic evolutionary relationships and classification. Additionally, a metabolomic study was conducted to gain deeper insights into this bacterium’s metabolic profile and functions. These results were essential for understanding the bacterium’s potential role in the autolysis phenomenon and its broader ecological significance.

The identification of 567 TRs and 28 CRISPR elements in the *L. xylanolytic* XL-2024 genome holds significant potential for genome stability and bacterial defense mechanisms. TRs are sequences where specific nucleotides are consecutively repeated, playing essential roles in genomic stability. These repeats can lead to replication fork stalling or errors during DNA replication, potentially resulting in genomic instability [32]. Additionally, TRs can modulate the transcription of adjacent genes by influencing the transcription factor binding [33]. In addition, the CRISPR system also contributes significantly to genomic stability by protecting bacteria from foreign DNA invasion and reducing the risk of mutations and rearrangements through limiting foreign DNA integration, thereby preserving genome integrity [34]. As an adaptive immune mechanism in bacteria and archaea, the CRISPR-Cas system recognizes and cleaves invading phages or plasmid DNA, shielding the host cells from infection [35,36]. Furthermore, CRISPR enhances the bacterial survival in competitive environments, bolstering fitness and adaptability [37].

Additionally, the *L. xylanilyticus* XL-2024 genome revealed a high abundance of “glycometabolic hydrolase”, “metallohydrolase hydrolase”, “lysozyme”, and “cell wall hydrolase”. These hydrolases are involved in various physiological processes in living organisms, including the hydrolysis of nucleotides and proteins, and RNA cleavage [38]. The body wall of sea cucumbers contains various bioactive compounds, including polysaccharides and peptides [39]. Given the activities of glucose metabolism hydrolase and metal hydrolase possessed by *L. xylanilyticus* XL-2024, it is plausible that this bacterium may contribute to the hydrolysis of sea cucumbers. In addition, cell wall hydrolases can break down the polysaccharides and proteins of the cell walls, thereby influencing their structure and function [40]. Moreover, lysozyme, with its primary antibacterial function, can break down bacterial cell walls, preventing bacterial growth and spread [41]. These results suggest that *L. xylanilyticus* XL-2024 possesses the ability to hydrolyze the cell walls of bacteria.

Additionally, genes linked to the Type III secretion system (T3SS) were present in the genome of *L. xylanilyticus* XL-2024, which are crucial for pathogenicity and symbiotic relationships with hosts. In pathogenic contexts, T3SS enables the injection of effector proteins directly into the host cells, modulating the signaling pathways and immune responses to facilitate pathogen invasion [42]. Certain T3SS-related genes also encode virulence factors that damage the host cells, leading to cell death, dysfunction, or inflammation [43]. In symbiotic relationships, T3SS assists pathogens in establishing stable associations with hosts; for example, specific plant pathogens deliver effector proteins to plant cells, triggering responses that support their survival [44]. T3SS genes further influence the host immune responses, promoting microbial persistence and stable symbiosis [45,46].

In addition, comparison and annotation with the GO database showed that the genes of the *L. xylanilyticus* XL-2024 genome mainly function in cellular and metabolic processes and participate in catalytic activities. Consistent with the GO enrichment results, the KEGG enrichment results revealed widespread annotations for amino acid metabolism, the metabolism of cofactors and vitamins, and carbohydrate metabolism. These results indicate that *L. xylanilyticus* XL-2024 may be able to degrade polysaccharides. Moreover, *L. xylanilyticus* XL-2024 exhibits a comprehensive and diverse metabolic pathway, facilitating the continuous exchange of substances and energy, further enabling the organism to maintain the resources necessary for its activities and respond promptly to environmental stimuli [47]. Additionally, the statistical analysis of genes in different categories in the Pfam database revealed that many genes were annotated as the “P-ring superfamilies containing nucleoside triphosphate hydrolases”. These genes function as ATPases, GTPases, and ATP synthases, playing important roles in the key enzymatic reactions [48]. Notably, the genes annotated in the VFDB database are not true virulence genes but rather regulatory genes that play significant roles in controlling biological processes. This suggests that *L. xylanilyticus* XL-2024 is safe and harmless in *A. japonicus*. The research on the adhesion and immune-modulating factors in the *Lysinibacillus* genus is limited; however, insights can be gained from the studies on *Bacillus* and *Paenibacillus* species, which share similar physiological and ecological traits with *Lysinibacillus*. For example, in the *Bacillus* genus, the adhesion factors of *Bacillus cereus* [49] and *Bacillus anthracis* have been widely studied, with surface proteins aiding these bacteria in colonizing and invading the host tissues to initiate infection [50]. Additionally, *B. anthracis* possesses multiple immune evasion factors, such as antiphagocytic proteins and toxins, which suppress the host immune responses [51]. *B. cereus* also produces toxins and immune-modulating proteins to evade the host defenses [52]. These studies suggest that *Lysinibacillus* species may similarly possess potential adhesion and immune modulation mechanisms.

A total of 86 CAZy genes were identified in *L. xylanilyticus* XL-2024 genome. Among these, 57 genes were associated with polysaccharide degradation, including CBM50, GH23, GH73, GH18, GH19, and GH24, which are mainly related to the degradation of crustacean shells and bacterial cell walls [53], and 29 genes were related to polysaccharide synthesis. The GH23 family mainly includes chitinase, lysozyme type G, and peptidoglycan lyase. Chitinases have garnered significant attention as biocontrol agents and are used to degrade fungal cell walls in producing fungal protoplasts [54]. Chitinases of the GH18 family are widely distributed across archaea, bacteria, fungi, higher plants, and animals [55]. In contrast, the chitinases of the GH19 family are predominantly found in plants and have a limited distribution in bacteria. The GH19 family chitinases in bacteria are believed to result from the horizontal gene transfer from plant chitinases. Furthermore, 13 genes related to various enzymes from the CBM32, CBM13, PL9_2, GH28, GH1, CE4, and CE9 families are involved in the degradation of plant cell walls [56]. Additionally, 26 genes associated with various enzymes from the GH23, GH73, GH18, GH19, and GH24 families primarily exhibit chitinase and lysozyme activities. Genes associated with CBM48 and CBM5 are linked to the degradation of other polysaccharides. The abundance of polysaccharide-degrading enzyme genes in the genome indicates that *L. xylanilyticus* XL-2024 has a robust capacity to effectively break down and utilize polysaccharides. Additionally, CAZymes play a crucial role in bacterial ecological adaptation and host interactions. In the gut, CAZymes enable bacteria to establish symbiotic relationships with their hosts by breaking down dietary polysaccharides, thus providing energy and enhancing nutrient absorption [57]. Furthermore, CAZymes facilitate the material exchange within microbial communities, increasing their stability and functionality, and allowing bacteria to efficiently utilize carbon sources and thrive in diverse environments [58].

Meanwhile, the gene family analysis revealed that *L. xylanilyticus* XL-2024 possesses 20 closely related strains, among which *B. amyloliquefaciens* XJ5 has been reported to have significant potential for hydrolyzing starch, *β*-glucan, and xylanase [59]. Previous studies have demonstrated that *B. amyloliquefaciens* produces *β*-1,3-glucanase and other proteins related to disease processes, which destroy fungal cell walls by targeting different components, thus resisting pathogenic fungal infections [60]. Moreover, *B. licheniformis* can secrete bacteriocin, an antimicrobial peptide, with strong antimycobacterial activity [61]. Furthermore, gene analysis identified 12 genes in the KEGG pathway annotation enriched in carbohydrate metabolism. The GO enrichment results revealed that two genes possess hydrolase activity acting on glycosyl bonds. Glycosyl hydrolases are a group of enzymes capable of hydrolyzing glycosyl compounds [62], such as glucose, galactose, and mannose, found in various living organisms, including tissues like cell membranes, extracellular matrix, bone, and muscles. These enzymes play crucial roles in processes such as energy metabolism, signal transduction, and cell recognition, as they break down glycosyl compounds into simpler forms like monosaccharides or oligosaccharides [63].

Notably, the metabolomics analysis of *L. xylanilyticus* XL-2024 revealed that various amino acids and their related substances were the most abundant metabolites in the positive ion mode, while several metabolites related to polysaccharide degradation were detected in the negative ion mode. Furthermore, specific metabolites such as propionic acid and ginkgolic acid were also identified. Propionic acid is known for its antibacterial properties and can contribute to the intestinal health of animals by aiding digestion, enhancing immunity, and inhibiting the growth of harmful microorganisms [64,65]. Previous studies have shown that adding propionic acid to poultry feed can increase the growth rate, improve the meat quality, and reduce the mortality [64]. Ginkgolic acid has been shown to inhibit the growth of *Mycobacterium tuberculosis* in vitro [65]. These properties indicate the potential of the *L. xylanilyticus* XL-2024 as a natural antimicrobial agent. In addition, carnosine, a small-molecule polypeptide and protein fragment, has been recognized as an important nutritional additive in feed and is widely used in the livestock and poultry industries [66]. It can enhance growth rates, and strengthen the immunity and effectiveness of antibiotics in animals, thereby boosting the economic benefits of farming [67]. Succinic acid and its derivatives also have great application potential in animal feed, which is crucial in improving the feed conversion ratio (FCR) and regulating the gut microflora in animals [68].

Additionally, various individual metabolites support bacterial survival and adaptation in the sea cucumber environment. For instance, glutamic acid and proline aid in nutrient acquisition and the stress response [69]. Fatty acids enhance the membrane fluidity and stability, facilitating bacterial adaptation to environmental changes [70]. Polysaccharides and glycosides are involved in energy metabolism and cell wall synthesis, which strengthen the bacterial resistance to external pressures [71]. Furthermore, antibiotics play a critical role in competition and defense [72]. These findings suggest that the metabolic processes in *L. xylanilyticus* XL-2024 are highly regulated and actively modulated. This regulation allows the organism to continuously exchange substances and energy and maintain the essential resources for its activities. Moreover, it equips *L. xylanilyticus* XL-2024 with the ability to degrade proteins and polysaccharides and provides it with antibacterial properties. Together, these metabolic capabilities enable the bacterium to effectively survive and adapt within the unique ecosystem of the sea cucumber habitat.

In the context of metabolic pathways, analyzing using the KEGG database revealed numerous genes enriched in amino acid metabolism, lipid metabolism, carbohydrate metabolism, arginine metabolism, and proline metabolism, observed in both the positive and negative ion modes. Additionally, some genes are also enriched in the metabolism of terpenoids and polyketides, the metabolism of cofactors and vitamins, and the biosynthesis of other secondary metabolites. The enrichment analysis of the KEGG pathways in bacterial genomes highlights their essential roles in ecological adaptation and medical applications. Symbiotic pathways, such as the metabolism of cofactors and vitamins [73], support the host nutrition, while pathogenic pathways, like lipid metabolism [74], enhance bacterial infectivity. Additionally, unique metabolic pathways—including the biosynthesis of other secondary metabolites [75], the metabolism of terpenoids and polyketides [76], and specific amino acid metabolism [77]—facilitate the bacterial adaptation to environmental changes and have potential applications in antimicrobial development and pollution remediation. These insights provide a valuable foundation for developing probiotics, antimicrobial agents, and ecological restoration technologies.

## 5. Conclusions

In summary, this study purified a unique bacterium, *L. xylanilyticus* XL-2024, from the hydrolysate of *A. japonicus*. Whole-genome analysis revealed that its genome size is 5,075,210 bp, with a GC content of 37.33%, encoding a total of 5275 genes. Functional annotations of the *L. xylanilyticus* XL-2024 genome indicated the presence of numerous hydrolases involved in glucose metabolism, such as metal hydrolases, lysozyme, cell wall hydrolases, and CAZymes. Moreover, significant enrichment was observed in the pathways related to amino acid metabolism, cofactor and vitamin metabolism, and carbohydrate metabolism. Gene family analysis further indicated that related strains possess substantial potential to hydrolyze starch, *β*-glucan, and xylan. Furthermore, specific functional genes enriched in the carbohydrate metabolism pathway demonstrated hydrolase activity, targeting glycosyl bonds. Metabolomics analysis also revealed that *L. xylanilyticus* XL-2024 produces several metabolites related to polysaccharide degradation, including peptidase, glucanase, and pectinase. Additionally, the detection of propionic acid and ginkgo acid in the metabolites may contribute to the stability of the *A. japonicus* hydrolysate. Together, these findings suggest that *L. xylanilyticus* XL-2024 may play a critical role in the autolysis of *A. japonicus*, while its antibacterial activity could help ensure the stability of the hydrolysate.

## Figures and Tables

**Table 1:** Statistics of the genome sequencing data for *L. xylanilyticus* XL-2024.

Overview of the Data	Number or %
Insert Size (bp)	350
Clean Reads Length (bp)	(150:150)
Raw Data (Mb)	1115
Filtered Reads (%)	10.34
Clean Data (Mb)	1000
Clean Data GC (%)	37.15
Clean Data Q20 (%)	97.29
Clean Data Q30 (%)	92

**Table 2:** Statistics of the K-mer analysis data for the genome of *L. xylanilyticus* XL-2024.

Genome Size Estimation	Number or %
K-mer	15
K-mer Number	265,881,182
K-mer Depth	53.59
Genome Size (Mb)	4.96
Revised Size (Mb)	4.87
Heterozygous Rat	0.02
Repeat Rate (%)	16.87

**Table 3:** Genome assembly summary for *L. xylanilyticus* XL-2024: scaffold and contig statistics.

	Scaffold	Contig
Total Num (>500 bp)	47	47
Total Length (bp)	5,075,210	5,075,210
N50 Length (bp)	217,930	217,930
N90 Length (bp)	78,763	78,763
Max Length (bp)	629,498	629,498
Min Length (bp)	970	970
Number of Gaps	0	0

**Table 4:** Statistical overview of the processed genome of *L. xylanilyticus* XL-2024.

Genetic Profile of the *L. xylanilyticus* XL-2024	Bp or %
Genome Size	5,075,210
Gene Number	5275
Gene Length	4,122,531
GC Content	37.33
% of Genome (genes)	81.23
Gene Average Length	782
Gene Internal Length	952,679
Gene Internal GC Content	34.13
% of Genome (internal)	18.77

**Table 5:** Statistics of the tandem repeat sequence results for the genome of *L. xylanilyticus* XL-2024.

Type	Number (#)	Repeat Size (bp)	Total Length (bp)	In Genome (%)
TR	567	3~374	56,515	1.1135
Minisatellite DNA	423	12~60	46,648	0.9191
Microsatellite DNA	13	3~6	525	0.0103

**Table 6:** Component analysis of the genome of *L. xylanilyticus* XL-2024.

Type	Number	Average Length (bp)	Total Length (bp)
ncRNA→tRNA	79	77	6119
5 s	12	115	1379
16 s	1	1540	1540
23 s	1	2925	2925
sRNA	160	68	10,995
Prophage	3	25,554.3	76,663
GIs	6	11,797	70,783
CRISPR	28	224.214	6278

**Table 7:** Statistics of the predicted CAZymes in the genome of *L. xylanilyticus* XL-2024.

CAZymes Family	Known Activity	Number
CBM50	Peptidases and amidases	26
CBM48	Glycogen-binding	1
CBM32	Binding to galactose	1
CBM13	Xylanase	1
CBM5	Binding to chitin	1
GH13_3	Pullulanase	4
GH23	Peptidoglycan lyase	3
GH73	Lysozyme	2
GH28	Polygalacturonase	2
GH18	Chitinase	2
GH24	Lysozyme	1
GH19	Chitinase	1
GH13_11	Pullulanase	1
GH13	Pullulanase	1
GH1	Lactase	1
GH0	Not yet assigned to a family	1
CE4	Chitooligosaccharide deacetylase	6
CE9	N-acetylglucosamine-6-phosphate deacetylase	1
PL9_2	Pectate lyase	1
GT4	Sucrose synthase	11
GT2	Cellulose synthase	8
GT51	Murein polymerase	4
GT26	*β*-glucosyltransferase	3
GT0	Not yet assigned to a family	2
GT28	*β*-N-acetylglucosaminyltransferase	1

## Data Availability

The dataset of the whole genome sequence reads presented in this study is available in an online repository, with the NCBI GenBank accession number PRJNA1135090.

## Supplementary Materials

The following supporting information can be downloaded at: https://www.mdpi.com/article/10.3390/foods13223662/s1, Figure S1. Gene annotation and functional classification of the genome of *L. xylanilyticus* XL-2024 based on TCDB. (a) TCDB primary subcategory statistical chart. (b) TCDB secondary subcategory statistical chart. Figure S2. The pfam annotation results of the genome of L. xylanilyticus XL-2024. Figure S3. Pan analysis of genome of *L. xylanilyticus* XL-2024 and 20 other similar type strains. (a) TCDB function classification of *L. xylanilyticus* XL-2024-specific genes. (b) Pathogenicity analysis of *L. xylanilyticus* XL-2024-specific genes. Figure S4. Metabolomic analysis of the culture of *L. xylanilyticus* XL-2024. (a) Total ion chromatogram (TIC) of groups in the positive model. (b) TIC of groups in the negative model. The lateral axis represents the retention time (ret. time). Table S1. The number of genes in the genome of *L. xylanilyticus* XL-2024 genome that are functionally annotated in various databases. Table S2. The main annotated hydrolases in the genome of *L.xylanilyticus* XL-2024 based on NR database. Table S3. The annotated virulence factors in the genome of *L.xylanilyticus* XL-2024 based on VFDB. Table S4. The number of metabolites superclass in the genome of *L. xylanilyticus* XL-2024.
