# Supplementary material for: Characterization of a Bacterium Isolated from Hydrolyzed Instant Sea Cucumber Apostichopus japonicus Using Whole-Genome Sequencing and Metabolomics
Source: Foods. 2024 Nov 17;13(22):3662. doi: 10.3390/foods13223662 (PMC11593622; doi:10.3390/foods13223662)

Supplementary Tables

Table S1. The number of genes in the genome of *L. xylanilyticus* XL-2024 genome that are functionally annotated in various databases.

Databases	Annotation_number
NR	4,759
KEGG	4,437
COG	3,298
GO	3,300
Pfam	3,300
SwissProt	2,146
TCDB	377
PHI	248
VFDB	192
ARDB	2
CARD	178
Secretory_Protein	103
T3SS	120
CAZy	86

Table S2. The main annotated hydrolases in the genome of *L.xylanilyticus* XL-2024 based on NR database.

Gene_id	Subject_description	Gene_number
GM002099	xylose isomerase	1
GM003765	vancomycin resistance protein	1
GM000006	tunicamycin resistance protein	1
GM000523	Tellurite resistance protein TerB	1
GM000995	sugar ABC transporter permease	1
GM002098	sugar ABC transporter ATP-binding protein	1
GM001408	sufurtransferase FdhD	1
GM004272	spore cortex-lytic enzyme	1
GM001714	saccharopine dehydrogenase	1
GM001026	RIP metalloprotease RseP	1
GM000916	resolvase	1
GM004327	pullulanase	1
GM001521	PTS sugar transporter subunit IIB	1
GM001522	PTS mannose transporter subunit IIA	1
GM001997	polysaccharide pyruvyl transferase	1
GM005221	peptidoglycan glycosyltransferase	1
GM004201	nitrate reductase	1
GM000976	metallophosphoesterase	1
GM001253	metallopeptidase	1
GM001255	metal-dependent hydrolase	1
GM002696	membrane-associated protease 1	1
GM004112	mannosyl transferase	1
GM000690	macrolide transporter	1
GM003063	lytic transglycosylase	1
GM002145	ion transporter	1
GM004270	glycosyltransferase	1
GM001892	fructose-bisphosphatase, class II	1
GM000558	fructose-2, 6-bisphosphatase	1
GM004347	Fosmidomycin resistance protein	1
GM004691	erythromycin esterase	1

GM004841	divalent metal cation transporter	1
GM004137	cell wall hydrolase	1
GM000302	arsenical-resistance protein	1
GM001748	arsenic transporter	1
GM000688	anion permease	1
GM001264	6-phosphofructokinase	1
GM000528	2-pyrone-4, 6-dicarboxylate hydrolase	1
GM000017	2-hydroxy-6-oxononatrienedioate hydrolase	1
GM000403	2-hydroxy-6-oxo-6-phenylhexa-2,4-dienoate hydrolase	1
GM001027	1-deoxy-D-xylulose-5-phosphate reductoisomerase	1
GM000993 GM001010	zinc protease	2
GM004580 GM004109	polysaccharide deacetylase	2
GM003748 GM002302	peptidoglycan hydrolase	2
GM001739 GM002569	nitroreductase	2
GM001191 GM003620	metal-binding protein	2
GM005159 GM002286	iron ABC transporter	2
GM000158 GM000811	glycosyl transferase family 2	2
GM004360 GM003654	fructokinase	2
GM001720 GM001443	1, 4-beta-xylanase	2
GM001001 GM004205 GM000747	Zn-dependent hydrolase	3
GM004728 GM005163 GM000160	oligosaccharide deacetylase	3
GM004658 GM000412 GM005014 GM001235	N-acetylmannosaminyltransferase	4
GM002593 GM000450 GM000777 GM003563	MBL fold metallo-hydrolase	4
GM004065 GM004694 GM004187 GM003726	HAD family hydrolase	4
GM002647 GM000383 GM004708 GM003689 GM003036 GM001629	permease	6
GM004915 GM003375 GM002935 GM000533	hydrolase	8

GM001691 GM001803<br>GM004989 GM002101<br>GM000899 GM000948<br>GM004505 GM000530<br>GM000484 GM000550<br>GM003386 GM003935<br>GM002601	alpha/beta hydrolase	9
GM002988 GM004519<br>GM000277 GM001503<br>GM002106 GM000824<br>GM003610 GM004864<br>GM000623 GM003803<br>GM004289 GM004583<br>GM000028 GM002429<br>GM002817 GM003576<br>GM000491 GM001369<br>GM003541 GM002988<br>GM000279 GM004642<br>GM004781 GM004526<br>GM000687 GM004873	MFS sugar transporter	26

Table S3. The annotated virulence factors in the genome of *L.xylanilyticus* XL-2024 based on VFDB.

Sequence	VF Name	Gene	VF category	Identity (%)
VFG043991	sphaericolysin (SPH)	Bsph_4094	—	89.4
VFG000077	ClpP	clpP	Stress survival	80.8
VFG046474	EF-Tu	OOM_0626	Adherence	77.9
VFG000079	ClpC	clpC	Stress survival	73.9
VFG016417	Polysaccharide capsule	gtaB	—	72.5
VFG019134	GroEL	groEL	Adherence	72.3
VFG037938	Capsule	M3Q_286	Immune modulation	72
VFG005579	Streptococcal enolase	eno	—	71.2

Table S4. The number of metabolites superclass in the genome of *L. xylanilyticus* XL-2024.

Mode	Superclass	Number
Positive ion mode	Organic oxygen compounds	141
	Organoheterocyclic compounds	63
	Lipids and lipid-like molecules	62
	Benzenoids	46
	Phenylpropanoids and polyketides	17
	Alkaloids and derivatives	7
	total	336
Negative ion mode	Organic acids and derivatives	74
	Lipids and lipid-like molecules	61
	Organoheterocyclic compounds	21
	Benzenoids	17
	Phenylpropanoids and polyketides	5
	Organic oxygen compounds	4
	Homogeneous non-metal compounds	2
	total	184

Supplementary Figures

Figure S1. Gene annotation and functional classification of the genome of *L. xylanilyticus* XL-2024 based on TCDB. (a) TCDB primary subcategory statistical chart. (b) TCDB secondary subcategory statistical chart.

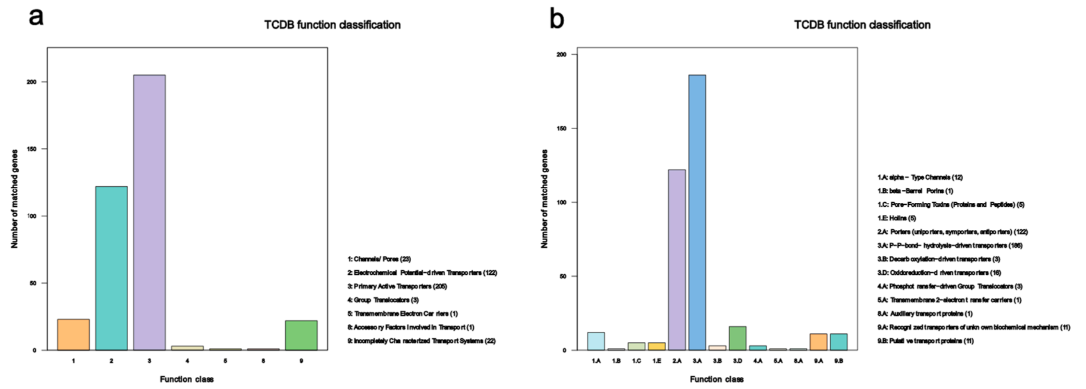

Figure S2. The pfam annotation results of the genome of *L. xylanilyticus* XL-2024.

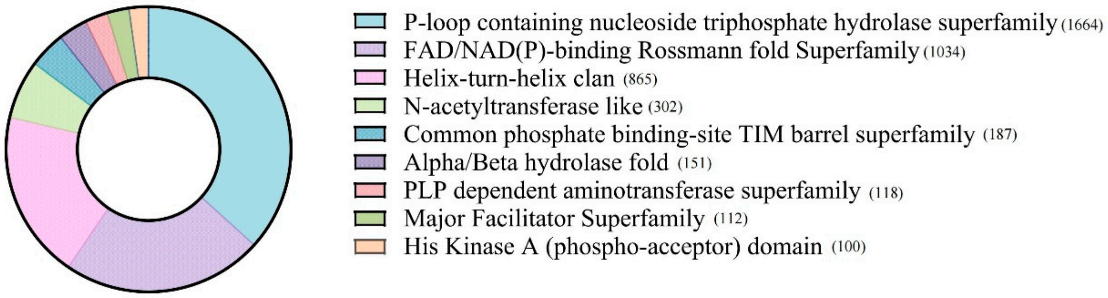

Figure S3. Pan analysis of genome of *L. xylanilyticus* XL-2024 and 20 other similar type strains. (a) TCDB function classification of *L. xylanilyticus* XL-2024-specific genes. (b) Pathogenicity analysis of *L. xylanilyticus* XL-2024-specific genes.

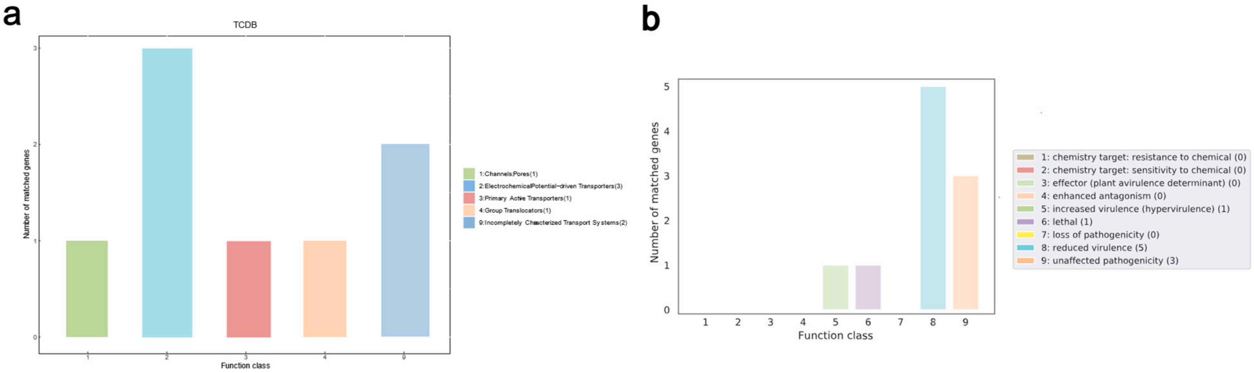

Figure S4. Metabolomic analysis of the culture of *L. xylanilyticus* XL-2024. (a) Total ion chromatogram (TIC) of groups in the positive model. (b) TIC of groups in the negative model. The lateral axis represents the retention time (ret. time).

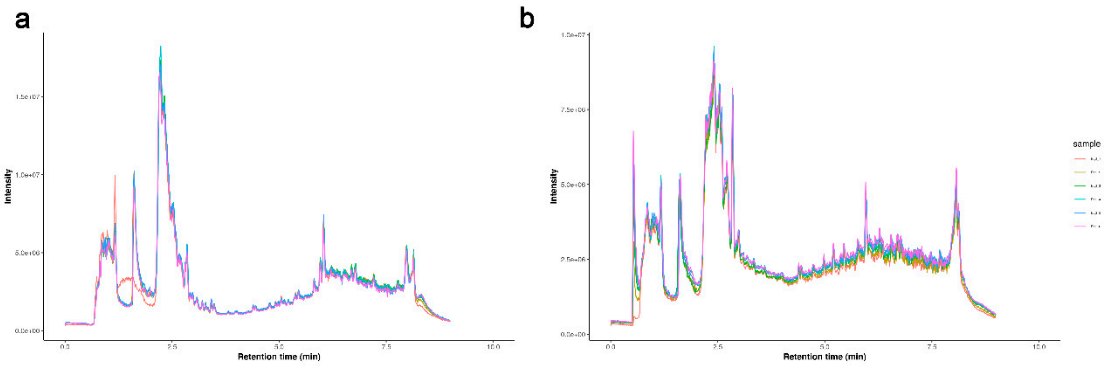

Supplement: Supplementary file 1 [file foods-13-03662-s001.zip › foods-3290428-supplementary.pdf]
